# Rare skeletal disorders: a multidisciplinary postnatal approach to diagnosis and management

**DOI:** 10.1007/s10354-021-00820-2

**Published:** 2021-03-10

**Authors:** Nina-Katharina Walleczek, Kristina Förster, Martina Seyr, Nadja Kadrnoska, Jennifer Kolar, Verena Wasinger-Brandweiner, Julia Vodopiutz

**Affiliations:** 1 Department of Pediatrics and Adolescent Medicine, Division of Pediatric Pulmonology, Allergology and Endocrinology, Comprehensive Center for Pediatrics, Medical University of Vienna, Vienna, Austria; 2 Department of Pediatrics and Adolescent Medicine, Division of Neonatology, Pediatric Intensive Care Medicine and Neuropaediatrics, Medical University of Vienna, Vienna, Austria; 3 Vienna General Hospital AKH, Vienna, Austria; 4 Vienna Bone and Growth Center, Vienna, Austria

**Keywords:** Whole exome sequencing in skeletal disorders, Pattern recognition, Vienna Bone and Growth Center, Diagnostic flow chart, Massively parallel sequencing, Vollständige Exomsequenzierung bei Skelettdysplasien, Mustererkennung, Zentrum für seltene Knochenerkrankungen Wien, Diagnostisches Flussdiagramm, Massive parallele Sequenzierung

## Abstract

Skeletal disorders are inherited disorders with significant skeletal involvement and most of them are rare or extremely rare. Based on the clinical, radiological and genetic phenotype, the group of skeletal disorder comprises more than 450 different and highly heterogeneous disorders. In skeletal disorders rapid and precise diagnoses are urgently needed for patient care and are based on the combination of clinical, radiological and genetic analysis. Novel genetic techniques have revolutionized diagnostics and have a huge impact on counseling of patients and families. Disease-specific long-term management in a multidisciplinary healthcare team in highly specialized centers is recommended to optimize care for these patients. Here we describe a multidisciplinary postnatal approach for the diagnosis and management of patients and families with rare skeletal disorders at the Vienna Bone and Growth Center. We discuss the value of a multidisciplinary diagnostic and management approach in the postnatal setting and provide a diagnostic flowchart for rare skeletal disorders.

## Abstract (de)

Skelettdysplasien zählen zur Gruppe der seltenen Krankheiten, wobei der Großteil der Skelettdysplasien zu den extrem seltenen Krankheiten gehört. Die Skelettdysplasien umfassen eine genetisch und klinisch äußerst heterogene Gruppe von über 450 unterschiedlichen angeborenen Erkrankungen, welche primär durch eine ausgeprägte Skelettbeteiligung charakterisiert sind. Anhand von klinischen, radiologischen und genetischen Merkmalen werden die Skelettdysplasien in Untergruppen klassifiziert. Eine rasche und akkurate Diagnosestellung der Skelettdysplasie ist die Voraussetzung für eine optimale medizinische Versorgung. Die Diagnosestellung der Skelettdysplasien basiert auf einer 3‑Säulen-Diagnostik mittels klinischer, radiologischer und genetischer Untersuchungen. Die Einführung neuer genetischer Untersuchungstechniken revolutionierte die Diagnostik der genetisch bedingten Skelettdysplasien und sind heute Teil der klinischen Routinediagnostik. Sie haben einen hohen Stellenwert für das therapeutische Management der Betroffenen sowie für die medizinische und genetische Beratung von Patient(inn)en und deren Familien. Zur Optimierung der Patient(inn)enversorgung wird eine krankheitsspezifische Behandlung in einem multidisziplinären Team in hochspezialisierten Zentren empfohlen. In dieser Arbeit wird der multidisziplinäre diagnostische und therapeutische Ansatz für die Betreuung von Patient(inn)en mit Skelettdysplasien und deren Familien am Zentrum für seltene Knochenerkrankungen „Vienna Bone and Growth Center“ skizziert. Die Autor(inn)en entwickelten ein diagnostisches Flussdiagramm zur postnatalen Abklärung von seltenen Skelettdysplasien und legen den Stellenwert einer multidisziplinären Betreuung der seltenen Skelettdysplasien dar.

## Introduction to rare skeletal disorders

Skeletal disorders are classified as inherited disorders with significant skeletal involvement resulting in abnormal bone length, density or shape. Most of them are rare—which is defined in Europe as less than 1 in 2000 people being affected—and some are extremely rare affecting only a tiny number of patients worldwide [1–3]. The current nosology and classification of skeletal disorders comprises more than 450 different skeletal diseases which are grouped in 42 different groups based on their clinical, radiological and/or genetic phenotypes [2].

The clinical phenotype in skeletal disorders is highly variable regarding severity, final body height and extraskeletal involvement. Severity in skeletal disorders ranges from perinatal lethality to very mild phenotypes such as premature degenerative joint disease or isolated mild short stature. Remarkable disproportionate short stature is a common feature in several skeletal disorders, but also normal stature and even tall stature is noted in some skeletal disorders [2, 4]. Extraskeletal involvement can be the clue to establish an accurate diagnosis [3, 4], such as cardiac defects (ciliopathies), cleft palate (type II collagenopathies; congenital glycosylation disorders with skeletal involvement), immunodeficiency and hematological abnormalities (cartilage hair hypoplasia; cell cycle defects, Fanconi anemia syndromes, Shwachman–Diamond syndrome), or severe myopia (type II collagenopathies) Table 1.

**Table 1:** Skeletal disorders: clinical and imaging workup and diagnostic clues

Major points	Clues examples
**Clinical work up to determine a clinical pattern**	
***Patient’s medical history***	
*Prenatal history*	
– Increased nuchal translucency	Skeletal disorders associated with increased nuchal translucency: – Achondrogenesis 2, some type II collagen disorders, ciliopathies, Greenberg dysplasia, every skeletal disorder with cardiac defects
– Growth parameters – Onset shortness long bones – Thoracic hypoplasia measured by thoracic circumference and by thoracic/abdominal ratio	Onset of shortness of long bones is usually earlier than onset of thoracic hypoplasia – thoracic hypoplasia is a caution signal for putative lethal skeletal disorders – normal thoracic measurements at 20 weeks gestational age do not exclude severe thoracic hypoplasia at birth Skeletal disorder associated with thoracic hypoplasia – Short-rib thoracic dysplasia, thanatophoric dysplasia, achondrogenesis 1A and 1B, some type II collagen disorders
– Fractures or bowing of bones	Skeletal disorder associated with prenatal fractures of long bones: – Osteogenesis imperfecta types II and III, hypophosphatasia, neurofibromatosis Skeletal disorder associated with prenatal bowing of long bones: – Symmetric: thanatophoric dysplasia, achondrogenesis types IA and IB, atelosteogenesis types I and II and III, campomelic dysplasia, diastrophic dysplasia, short-rib thoracic dysplasia – Asymmetric: osteogenesis imperfecta types II and III, hypophosphatasia
– Hypomineralization	Skeletal disorder associated with hypomineralization – Osteogenesis imperfecta: hypomineralization of the facial bones and skull with normal mineralization of hands, platyspondyly, fractures, curved long bones – Hypophosphatasia: hypomineralization especially of hands, fractures and chromosome-like long bones – Campomelic dysplasia: hypoplastic fibula and scapula, facial dysmorphism, malformations – Cleidocranial dysplasia: hypo- or aplastic claviculae, hypomineralization of skull, normal long bones – Achondrogenesis IA: hypomineralization of skull and spine, rib fractures, severe micromelia
– Polyhydramnion	– Risk factor for prematurity; Might be a caution signal for clinical relevant foramen magnum stenosis in achondroplasia
– Malformations and fetal profile	Characteristic facial features in skeletal disorders: – frontal bossing, midface hypoplasia, nasal flattening Skeletal disorders with cardiac defects especially left side cardiac defects: – e.g., ciliopathies like short-rib thoracic dysplasia, Ellis-van Creveld syndrome, orofaciodigital syndrome Within the group of lethal skeletal disorders – Cloverleaf skull malformation: thanatophoric dysplasia – Microretrognathia, cleft palate: some type II collagen disorders – Polydactyly and cardiac defects: ciliopathies such as short-rib thoracic dysplasia
– Maternal factors (illness, medication, alcohol, …)	– Fetal warfarin or thalidomide syndrome – Fetal akinesia sequence
– Prenatal genetic investigations	– Is there a genetic diagnosis? If yes does this diagnosis correlate with the postnatal clinical and radiological pattern? Which disorders have been excluded genetically?
*Growth parameters and* *percentiles*	– From birth to current age – Use disease-specific percentiles whenever available
*Complete medical and* *developmental history*	– Severe illness; pain; progression; – Evidence for extraskeletal manifestation, developmental delay or intellectual disability
*Family history and pedigree* *analysis*	– To establish mode of inheritance: draw at least a 3-generation pedigree and a 5-generation pedigree in case of consanguinity. Similar affected family members? Miscarriages, still born? Known diseases in the family
***Patient’s physical examination***	
*Growth parameters*	– Height; weight; head circumference; armspan; sitting height; upper/lower segment ratio
*Skeletal disorder-specific* *physical examination*	
– Stature – Assessment of asymmetry or symmetry	– Stature: short/normal/tall and proportionate/disproportionate – Short limbs: transversal limb defects, rhizomelia, mesomelia, acromelia, micromelia – Type of brachydactyly; narrow chest; scoliosis – Symmetry or asymmetry: Asymmetric involvement with genetic cause: X‑linked dominant chondrodysplasia punctata‑2, Russel Silver syndrome, segmental overgrowth syndromes, neurofibromatosis Asymmetric involvement with exogenic cause: limb defects due to amniotic bands or due to vascular disruption defects
– Facial dysmorphism	If yes? Characteristic facial features pointing to a specific diagnosis such as – Russel Silver syndrome, 3M syndrome, craniosynostosis syndromes, progeria
– Fractures	– Hypophosphatasia, osteogenesis imperfecta, osteopetrosis versus adequate trauma
– Heterotopic ossifications	Skeletal disorders with heterotopic ossifications: – Fibrodysplasia ossificans progressiva, primary osteoma cutis, progressive osseous heteroplasia, pseudohypoparathyroidism, Albright’s hereditary osteodystrophy, pseudopseudohypoparathyroidism
– Connective tissue involvement/dislocations; contractures/joint limitations	Skeletal disorders with hyperlaxity – several including: collagenopathies, GAG-biosynthesis disorders, cartilage hair hypoplasia, *NEPRO*-associated skeletal disorder, pseudoachondroplasia
– Hair, nails and teeth	Skeletal disorders with ectodermal involvement – Sparse hair: cartilage hair hypoplasia, trichorhinophalangeal syndrome, *NEPRO*-associated skeletal disorder – Nail abnormalities: nail–patella syndrome – Dental anomalies and nail hypoplasia: Weyers acrofacial dysostosis, Ellis-van Creveld – Dentigonesis imperfecta or hypoplasia of enamel: osteogenesis imperfecta, hypophosphatasia – Natal teeth: some progeria syndromes, Ellis-van Creveld, – Supernumerary teeth: cleidocranial dysplasia, Ellis-van Creveld, Weyers acrofacial dysostosis – Hyperpigmentation and café au lait spots: cell cycle defects, neurofibromatosis
– Malformations and organ dysfunctions	– Cleft palate: type II collagenopathies, GAG-biosynthesis disorders, hyperphosphatasia with mental retardation syndrome – Oral frenulae: ciliopathies such as Ellis-van Creveld – Severe myopia: type II collagenopathies – Infantile glaucoma: type II collagenopathies, GAG-biosynthesis disorders, nail–patella syndrome, filamin A disorders, oculoskeletodental syndrome – Risk for retinopathy, nephropathy and hepatopathy: ciliopathies – Cataracts: X‑linked dominant chondrodysplasia punctata‑2, inborn errors of metabolism with skeletal disorders – Gastrointestinal malformations: cartilage hair hypoplasia, hyperphosphatasia with mental retardation syndrome – Risk for immunodeficiency or hematological involvement: cartilage hair hypoplasia, cell cycle defects, Fanconi anemia syndromes, Shwachman–Diamond syndrome
– Visual diagnostic clues to rare skeletal disorders	– Congenital malformation of halluces, painful swelling of soft tissue, progressive heterotopic ossification: fibrodysplasia ossificans progressiva – Cystic swelling of the ear: diastrophic dysplasia – Dental and nail abnormalities, mild short stature, polydactyly, ear dysplasia: Weyer acrofacial dysostosis – Absent or hypoplastic patella: nail–patella syndrome – Painful, fleshy papules, progressive contractures and diarrhea: hyaline fibromatosis syndrome – Acanthosis nigricans: thanatophoric dysplasia, achondroplasia, hypochondroplasia
– Caution signals	– Respiratory insufficiency/failure due to upper airway obstruction or small chest; neurological abnormalities due to foramen magnum stenosis or cervical spine instability; hearing loss; severe infections
*General physical and* *laboratory examination*	– To exclude relevant multisystemic involvement and malformations
***Family investigation***	
*Growth parameters* *Skeletal disorder-specific* *physical examination* *Dysmorphic examination*	– Height; weight; head circumference; armspan; sitting height; upper/lower segment ratio – Define if other family members are affected and if dysmorphic features in the patient might be familial variants
**Imaging workup to determine an imaging pattern**	
*Complete skeletal survey* – Spine ap. and lat. – Pelvis with hips ap. – Both hands and feet ap. – Long bones ap. unilateral (bilateral in asymmetric involvement) – Skull lat. – Consider special radiographs dependent on clinical manifestation: radiographs for fractures, flexion and extension of cervical spine	Define a radiological pattern – Which part of long bones: epi-/meta-/diaphyseal – Spine affected? – Which part of the bones are affected/non affected? Don’t miss disease characteristic signs such as – iliac horns in nail–patella syndrome; hypoplastic iliac wings, trident acetabular roofs and sciatic notch spur in ciliopathies; snail-like appearance of the ilia in Schneckenbecken dysplasia; hypoplastic or aplastic claviculae and large fontanells in cleidocranial dysplasia; double-layered patella in multiple epiphyseal dysplasia; Define bone age – Retarded bone age is common in skeletal disorder while accelerated bone age is rare and links to GAG-biosynthesis disorders – Bone age is not suitable to estimate adult height in skeletal disorder Consider age dependent radiological patterning: – For example, chondrodysplasia punctate and accelerated bone age are vanishing with time; in adults: try to obtain pediatric skeletal radiographs
*Consider disease-specific imaging* – e.g., MRI, CT, DXA ultrasound, SPECT, HR-pQCT	Don’t miss important complications of skeletal disorders such as – Foramen magnum stenosis in achondroplasia; cervical canal stenosis in heterotopic ossification disorders, chondrodysplasia punctate brachytelephalangic; cervical spine instability for example in SEDC, GAG-biosynthesis disorders, SMED

*CNV* copy number variants, *CT* computed tomography, *DXA* bone densitometry, *GAG* glycosaminoglycan, *HR-pQCT* high resolution peripheral quantitative computed tomography, *MRI* magnet resonance imaging, *PA* panel analysis, *SED* spondyloepiphyseal dysplasia, *SMED* Spondylo-meta-epiphyseal dysplasia, *SNV* single nucleotide variants, *SPECT* single-photon emission computed tomography

**Fig. 1:**
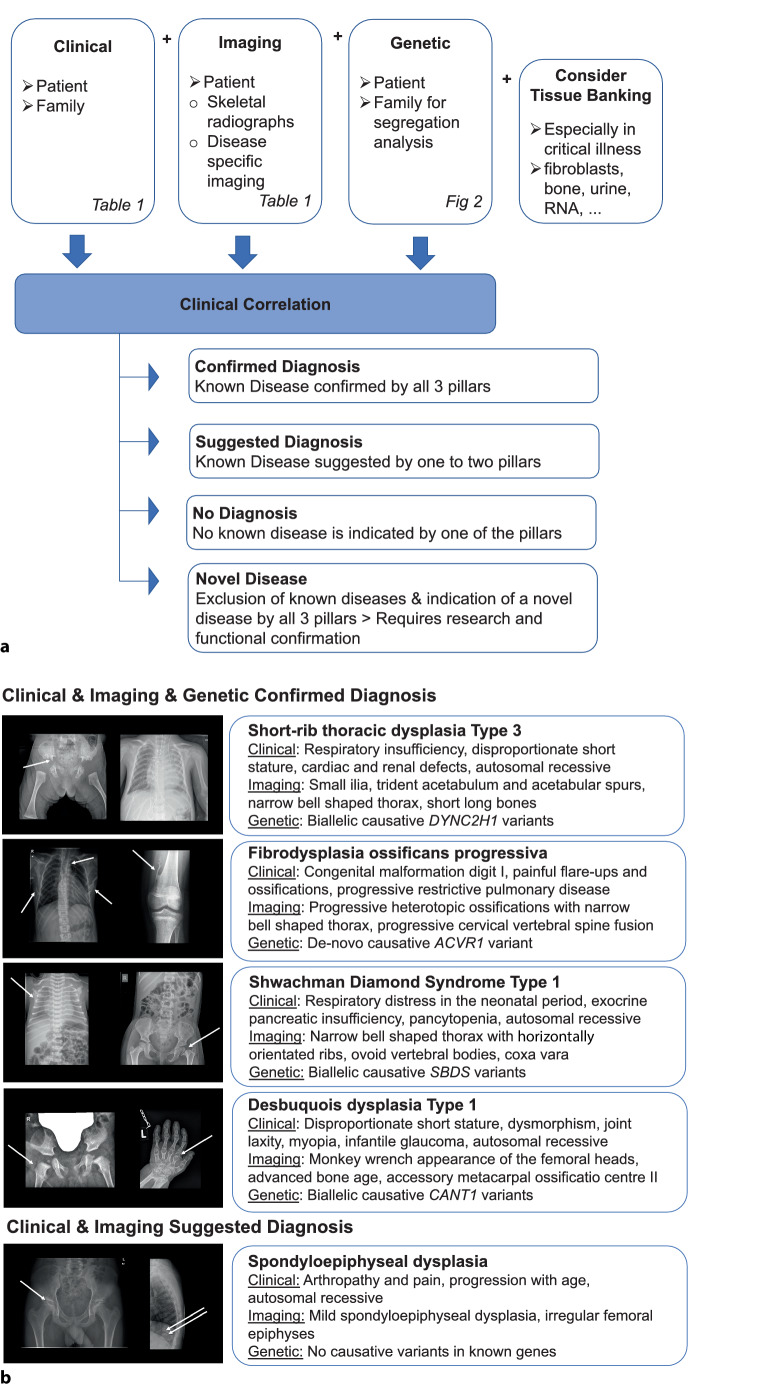
Establishment of diagnosis in rare skeletal disorders using the three pillar approach. **a** Clinical, imaging and genetic testing is followed by clinical correlation to establish a diagnosis. The main goal is to establish a diagnosis based on correlation of all three pillars. Tissue banking, at least banking of fibroblasts, is strongly recommended in all critically ill patients and is suggested in patients with negative or inconclusive genetic testing or potential novel diseases to facilitate functional confirmation. **b** Examples for clinical, imaging and genetic pattern recognition in well-known skeletal disorders

In addition to clinical heterogeneity, the molecular spectrum of skeletal disorders is very broad. The more than 430 skeletal disorder causing genes are functionally diverse, affecting critical steps in bone and cartilage development as well as extraskeletal functions such as regulating metabolic pathways, cell division, gene transcription, or intracellular trafficking. The detailed function of several skeletal- disorder-causing genes has not been elucidated yet [2]. Within one and the same gene, different pathogenic variants can cause different skeletal disorder phenotypes: variants in the *COL2A1 *gene cause more than ten different type II collagenopathy phenotypes, ranging in severity from severe perinatal lethality to isolated premature arthrosis in adults [5]. On the other hand, one skeletal disorder phenotype can be caused by pathogenic variants in several different genes: ciliopathies with major skeletal involvement are clinically characterized by a narrow thoracic cage, short and horizontally layered ribs, shortened tubular bones and by a characteristic radiological patterning on skeletal radiographs of the pelvis (Fig. 1) and can be caused by pathogenic variants in more than 20 different genes [6, 7]. In addition, a large number of rare genetic syndromes present with prenatal or postnatal onset of short stature but without significant skeletal involvement [8], which adds complexity to the diagnostic approach in skeletal disorders.

For all these reasons making an accurate diagnosis in skeletal disorders can be challenging. However, for clinical care of patients with skeletal disorders, it is crucial to establish a rapid and precise diagnosis to offer a disease-specific multidisciplinary therapeutic approach [2, 4, 9].

Here we describe the postnatal approach to diagnosis and management of patients and families with rare skeletal disorders by the multidisciplinary team of the Health Care Provider 1 (HCP1) at the Vienna Bone and Growth Center.

## Combined clinical, radiological and genetic diagnostic approach

The diagnosis of skeletal disorders is usually based on a three pillar system including clinical, imaging and genetic analyses [2–4, 10–12]. The main goal of the diagnostic approach is to establish a clinical, imaging and genetic confirmed diagnosis of the skeletal disorder, to offer disease-specific patient management as well as disease-specific medical and genetic counseling to patients and families (Fig. 1a).

**Fig. 2:**
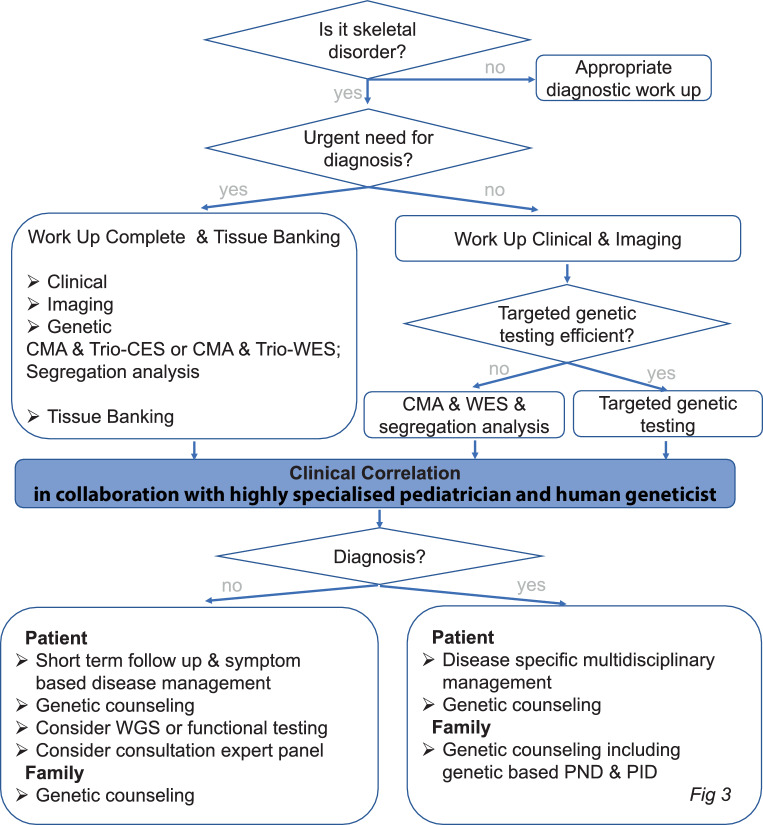
Postnatal diagnostic flow chart for skeletal disorders. Need for special disease management and monitoring for treatable complications should be evaluated at every patient contact and should not be postponed until the result of genetic testing is available. Need for urgent testing: critically ill patients, if results of genetic testing will influence the therapeutic management, pregnancy in patients or in a potential carrier. Trio genetic testing is preferred whenever possible to increase the chance of finding a definitive diagnosis and better interpretation of results. Targeted testing may be efficient when the clinical and radiological patterns indicate a very specific diagnosis and causative genes are not too large (e.g., achondroplasia), when a familial disease-causing variant is known, or when certain variants are more common in a population. *CES* clinical exome sequencing, *CMA* chromosomal microarray analysis, *PID* pre-implantation diagnostic, *PND* prenatal diagnostic, *RNA* ribonucleic acid, *WES* whole exome sequencing, *WGS* whole genome sequencing

We established a systematic approach for the postnatal diagnostic workup of skeletal disorders, which is outlined in Fig. 1a and 2. It requires a detailed patient history, careful clinical examination of the patient and their family, as well as a complete skeletal radiographic evaluation (Table 1) to determine the clinical and radiographic pattern in skeletal disorders (Fig. 1b). Familiarity with skeletal disorders and other rare pediatric diseases as well as good pattern recognition skills are mandatory for establishing an accurate clinical and radiological diagnosis.

Genetic testing is the third diagnostic pillar in the diagnostic approach of skeletal disorders. Novel genetic techniques, such as massively parallel sequencing technologies, have revolutionized diagnostics in skeletal disorders and have had a huge impact on counseling of patients and families as well as on therapeutic decision making [2, 9]. Thus, being familiar with these novel genetic techniques is important for state-of-the-art patient care and should be available at highly specialized centers [9].

Massively parallel sequencing technologies allow extremely fast sequencing of the whole exome (whole exome sequencing, WES) or of targeted parts of the exome (clinical exome sequencing CES; panel analysis PA) in a single assay in the patient (mono-investigation) or in the patients and their parents (trio-investigation). Thereby detection of disease-causing variants—including single nucleotide variants (SNVs), small insertion or deletion (Indel), and copy number variants (CNVs)—is fast and highly cost-effective [13]. In addition, chromosomal microarray (CMA) is applied to exclude unbalanced chromosomal aberrations. In the near future, novel techniques, such as whole genome sequencing (WGS), will presumable replace CMA, CES, PA and WES due to diagnostic superiority [2, 14].

In recent years, these novel genetic techniques have become the first-tier diagnostics for rare monogenetic phenotypes and therefore have replaced a detailed clinical investigation and more invasive diagnostic procedures in many medical disciplines [14]. In contrast interpretation of genetic data in skeletal disorders often requires detailed clinical and radiological phenotyping, implying a combined clinical, radiological and genetic workup as the best first-tier diagnostic approach in skeletal disorders [2, 12]. Even in skeletal disorders which can be diagnosed based only on clinical or radiological patterning, genetic confirmation is recommended, as identification of the disease-causing variant is mandatory for therapeutic decisions (e.g., osteogenesis imperfecta) and molecular prenatal and preimplantation diagnostics (PND, PID) (e.g., Schneckenbecken dysplasia or ciliopathies) [9].

**Fig. 3:**
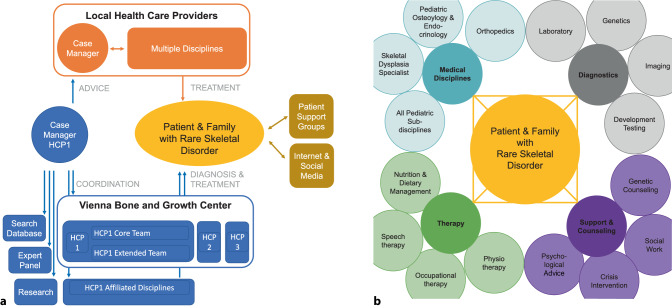
Postnatal multidisciplinary management for skeletal disorders. **a** Management of rare skeletal disorders at the HCP1 Vienna Bone and Growth Center. Patient support groups and social media are an additional important source for many patients and their families. **b** Skeletal disorders affect skeletal and variable extraskeletal systems. Optimal management requires a disease-specific multidisciplinary team approach. *HCP* health care provider

The key step within the diagnostic approach to skeletal disorders is the clinical correlation of clinical, imaging and genetic data to define a final diagnosis. Within the working group for rare skeletal disorders and unknown syndromes at the Vienna Bone and Growth Center, this is established in close collaboration of a highly specialized pediatrician and a human geneticist (Fig. 2). Sometimes rare and extremely rare skeletal disorders require consultation with an international expert panel to establish an accurate diagnosis (Fig. 2 and 3).

## Multidisciplinary management approach

It is strongly recommended that patients with rare skeletal disorders should be managed in highly specialized centers, such as the European Reference Network on rare bone diseases [15]. This should improve access to high-quality healthcare for patients and their families and should facilitate the generation of reliable data on rare skeletal disorders in order to improve further treatment strategies [9, 15]. In recent years, specific treatments have been developed for a small number of skeletal disorders [9, 16], but for the majority of skeletal disorders current treatment options consists of symptomatic approaches and in monitoring for treatable complications [2, 9].

Skeletal disorders are associated with a highly variable range of clinical and nonclinical challenges for patients, families and health care providers and therefore need a multidisciplinary management approach [2, 9, 17]. The multidisciplinary management approach for skeletal disorders and their families at the HCP1 Vienna Bone and Growth Center is outlined in Fig. 3. Additional care by a multidisciplinary team close to the patient’s home (local health care provider) is desirable, especially if the patient lives far away from the Vienna Bone and Growth Center. Multidisciplinary management of rare skeletal disorders requires coordination of diagnostic and therapeutic issues for each patient. For coordination issues one specialized pediatrician from the HCP1 Vienna Bone and Growth Center, who is experienced in managing this disorder, is defined as the primary case coordinator for each patient (medical case manager). This case manager is responsible for providing advice regarding local health care providers for local disease-specific management, to coordinate patients’ diagnostic and therapeutic approaches within the Vienna Bone and Growth Center, to perform database searches, and—if necessary—to communicate with expert panels and research groups (Fig. 3a). The multidisciplinary team for skeletal disorders at the Vienna Bone and Growth Center includes all disciplines needed for patient care. Besides access to all medical subdisciplines, to several diagnostic techniques and to specialized therapies (e.g., physio-occupational and speech therapy, and nutrition and dietary treatment to prevent obesity), it is important to offer social work, genetic counseling, psychological advice and crisis intervention to patients and families (Fig. 3b).

Furthermore, it is important to remember that suffering from a rare disorder often starts with a diagnostic odyssey, followed by persistent and significant burden to patients and their families [17]. A multidisciplinary management approach in highly specialized centers should be directed by high-quality healthcare as well as by awareness that patients and their families need constant multidisciplinary support to cope with medical, psychological, social, financial and further burdens of rare diseases.

## Conclusion

Skeletal disorders comprise a very heterogeneous group of rare and extremely rare genetic disorders with major skeletal involvement. Standardized diagnostic and management approaches in highly specialized centers are recommended for optimal patient care.
